# Genes on B chromosomes of vertebrates

**DOI:** 10.1186/s13039-014-0099-y

**Published:** 2014-12-17

**Authors:** Alexey I Makunin, Polina V Dementyeva, Alexander S Graphodatsky, Vitaly T Volobouev, Anna V Kukekova, Vladimir A Trifonov

**Affiliations:** Institute of Molecular and Cellular Biology SВ RAS, Novosibirsk, 630090 Russia; Theodosius Dobzhansky Center for Genome Bioinformatics, St. Petersburg State University, St. Petersburg, Russia; Novosibirsk State University, Novosibirsk, Russia; Museum National d’Histoire Naturelle, Origine, Structure et Evolution de la Biodiversite, Paris, France; Department of Animal Sciences, The University of Illinois at Urbana-Champaign, Champaign, USA

**Keywords:** B chromosomes, Segmental duplication, Proto-oncogenes, Vertebrates, Evolution of genomes

## Abstract

**Background:**

There is a growing body of evidence that B chromosomes, once regarded as totally heterochromatic and genetically inert, harbor multiple segmental duplications containing clusters of ribosomal RNA genes, processed pseudogenes and protein-coding genes. Application of novel molecular approaches further supports complex composition and possible phenotypic effects of B chromosomes.

**Results:**

Here we review recent findings of gene-carrying genomic segments on B chromosomes from different vertebrate groups. We demonstrate that the genetic content of B chromosomes is highly heterogeneous and some B chromosomes contain multiple large duplications derived from various chromosomes of the standard karyotype. Although B chromosomes seem to be mostly homologous to each other within a species, their genetic content differs between species. There are indications that some genomic regions are more likely to be located on B chromosomes.

**Conclusions:**

The discovery of multiple autosomal genes on B chromosomes opens a new discussion about their possible effects ranging from sex determination to fitness and adaptation, their complex interactions with host genome and role in evolution.

## Introduction

Supernumerary chromosomes were first described by Wilson [1] in the bug of the genus *Metapodius*. Later dispensible karyotypic elements were also found in rye and maize [2-4] and Randolph [5] suggested the term “B chromosomes” in 1928 to underline the nonessential nature of these elements. Since then B chromosomes (or supernumeraries, extra chromosomes, or Bs) have been found in all major eukaryotic clades. In mammals, where the majority of about 5000 known species are karyotyped, B chromosomes were reported for 75 species ([6], our data).

The variation in number of Bs between populations, individuals or even cells and tissues within an individual might be explained by their mitotic instability and existence of specific accumulation mechanisms acting during process of gametogenesis. Early works on mammalian B chromosomes accumulated data from different species and compared them with data on supernumerary chromosomes of invertebrates and plants, resulting in the idea that B chromosomes represent a system complementary rather than competitive to autosomes [7].

Different theories were proposed regarding B chromosome origin and dispersal within populations. Usually it was hypothesized that B chromosomes originate from autosomes of host species or result from interspecific hybridization (see [8,9] for review). An interesting theory postulates that Bs occur more frequently in genomes with more acrocentric chromosomes due to the centromeric drive in female meiosis [10].

In contrast to other supernumerary karyotype elements (*e.g.,* double minutes [11] or small supernumerary marker chromosomes [12]), B chromosomes are somehow preserved in host species over many generations and spread over many individuals within populations. The reason for their stability may lie in the B chromosome genetic content. In this case, evidence of enrichment with some genomic elements in Bs would shed light on the mechanisms of their prevalence and stability in the populations of some (but not all) species.

Traditionally, B chromosomes were considered to be totally heterochromatic (i.e. transcriptionally inactive) or, if they had evolved recently, to be undergoing the heterochromatization process according to Muller’s ratchet mechanism. Indeed, the heterochromatic nature of B chromosomes agrees with the fact of their high variability, and these elements often demonstrate typical C-positive staining, although C-negative B chromosomes also exist in mammals [13-15].

## Review

### Molecular structure of B chromosomes

Early cytogenetic studies of B chromosome molecular composition used available repetitive probes to find specific sequences localized on Bs. These could be tandemly arranged repetitive elements [16], LINEs (long interspersed nuclear elements) and SINEs (short interspersed nuclear elements) [17], interstitial telomeric sequences [18,19], ribosomal DNA clusters [20,21], or histone genes [22]. These studies confirmed the presence of different repetitive elements (also occurring in the standard genome) on Bs in all studied species. Further studies of B chromosomes involving B chromosome isolation by either flow sorting or microdissection followed by reverse painting failed to provide information about any homologous regions between autosomes and B chromosomes [23-25]. Only localization of BAC (bacterial artificial chromosome) clones and sequencing of isolated B-specific DNA fragments led to the identification of non-repetitive sequences on Bs [25]. The application of these techniques significantly changed our view of the molecular composition of supernumeraries.

With the development of high throughput sequencing technologies, the rapid growth of genomic sequence data provides new insights into chromosomal organization in all branches of the tree of life. In vertebrates, most comprehensive studies have been performed on mammals. Over 95% of DNA sequence is assembled into chromosomes for human, the remainder being mostly satellite DNA in centromeric and pericentromeric regions. Regularities of genome organization were discovered, such as the abundance of “gene islands” and “gene deserts”, the proportion and distribution of various repeat families, and the presence of ultra-conserved elements and long non-coding RNAs in non-coding regions [26-28]. The application of novel methods of high throughput sequencing, coupled with a growing knowledge of genome organization, together open a new chapter in B chromosome studies.

## Genes on B chromosomes of canids

Canids represent an outstanding order of mammals in terms of chromosomal evolution rates [29]. Additionally, a higher number of canid species with B chromosomes has been reported in comparison to other mammalian groups. B chromosomes were observed in genomes of red, Bengal and pale foxes (*Vulpes vulpes, V. bengalensis* and *V. pallida*) [30-32], Japanese and Chinese raccoon dogs (*Nyctereutes procyonoides procyonoides* and *N. p. viverrinus*) [33,34], the short-eared dog (*Atelocynus microtis*) [35] and the maned wolf (*Chrysocyon brachyurus*) [36]. Among B-carrying canids, the most intensive karyotype studies were accomplished in red foxes and raccoon dogs [29,30,33,34,37]. In the 1970s, the B chromosomes of the red fox were thoroughly studied to detect number variation patterns between and within individuals [30], late replication timing [38], and preferential segregation towards reproductive cells [39]. Classical cytogenetic studies revealed clusters of inactive ribosomal genes, interstitial telomeric sequences, and complex chromatin structure in raccoon dog B chromosomes [18,19]. Later, B chromosomes of red foxes and raccoon dogs were isolated by microdissection [25] and flow sorting ([24] and our unpublished data) and the resulting B specific DNA libraries were used in reverse painting FISH experiments.

The first autosomal gene – *C-KIT* (v-kit Hardy-Zuckerman 4 feline sarcoma viral oncogene homolog) - was discovered by chance on red fox B chromosomes during the fox genome mapping project using canine BAC clones. Later the same gene was discovered on B chromosomes of Chinese and Japanese raccoon dogs [40]. Autosomal *C-KIT* contains 21 exons [41] and encodes a transmembrane tyrosine kinase regulating proliferation and cell differentiation of melanoblasts, blood cells, and primordial germ line cells [42]. The RNA coding region (1100 bp) of the *C-KIT* gene is homologous to provirus of feline Hardy-Zukerman sarcoma 4 [43]*.* Mutations in *C-KIT* can cause gut stromal cancers in human, mouse, dog and rat [44]. Many mutations identified in *C-KIT* lead to a white spotting pattern of coat color [45]. While this pattern is widely distributed across mammals, its causative mutations vary: these can be missense substitutions [45-47], exon skipping [48], duplication combined with splice mutations [49] or retrovirus insertions [50]. Detection of BAC signals on canid B chromosomes might also be caused by the presence of rather small but highly amplified unique segments. To exclude this possibility, the *C-KIT*-containing region was analyzed using PCR-assisted mapping and sequencing. The autosomal fragment on fox B chromosomes was found to encompass at least 480 kbp and to include the *C-KIT* gene, the pseudogene *RPL23A* and a large intergenic spacer on both the 3’ and 5’ gene flanking regions. The fragment of the raccoon dog B chromosomes was found to be even larger and to contain at least 490 kbp including *C-KIT*, the pseudogene *RPL23A*, part of the *KDR* (kinase insert domain receptor) gene and a large intergenic spacer on the 5’ end of *C-KIT* [51].

**Figure 1:**
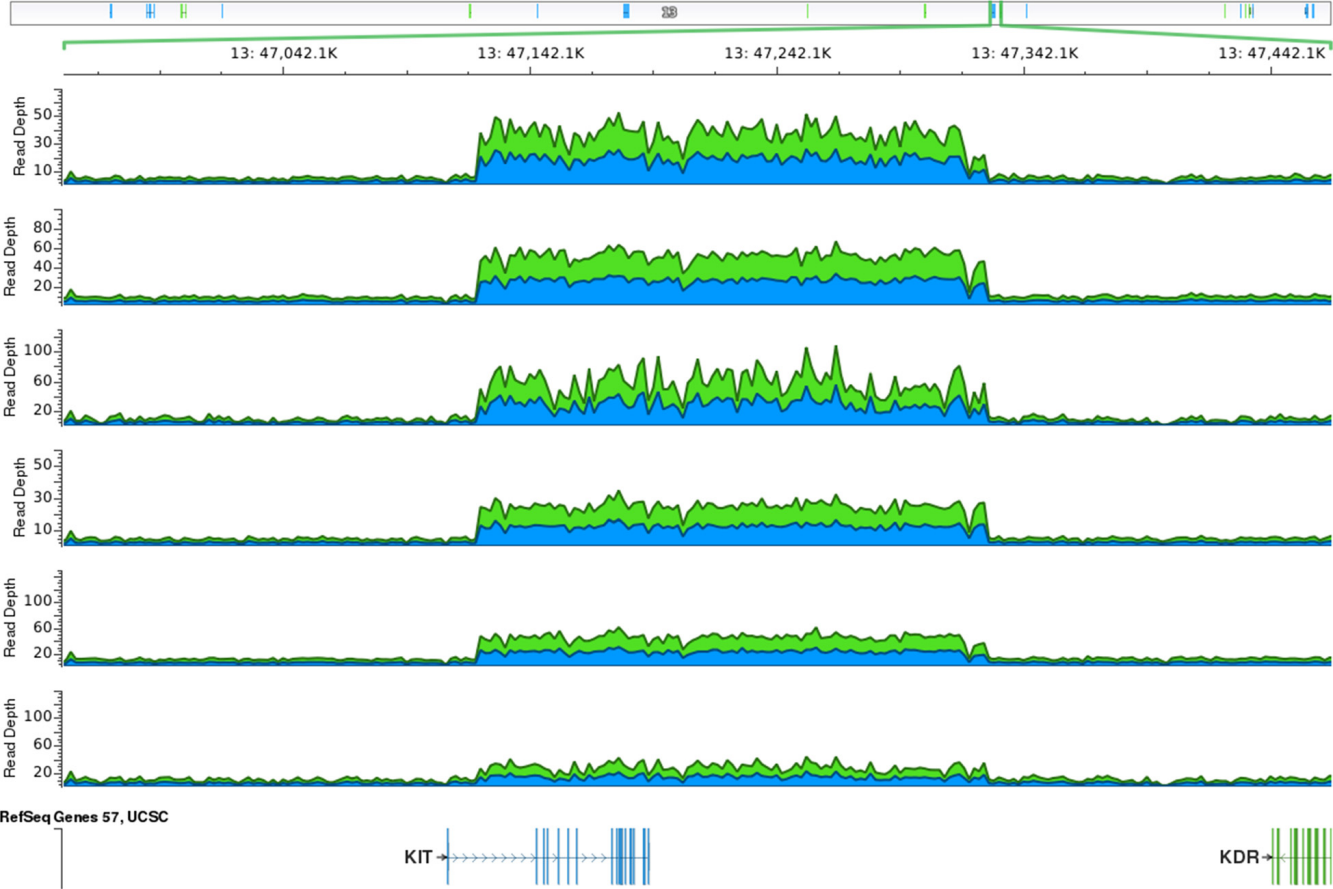
**Visualization of the fox read alignment to a region of the dog chromosome 13 containing** ***C-KIT*** **gene with GoldenHelix GenomeBrowse 1.1.2.** Alignment of Illumina reads from each of the six sequenced foxes showed an increase in the sequencing depth over a ~ 206 kb region. This region includes a part of intron 1 and exons 2–21 of the *C-KIT* gene [48]. The depths of the read increase vary among the six foxes suggesting that sequenced individuals carry different numbers of B chromosomes in their genomes.

Some new data on the *C-KIT* containing segment came from the fox genome sequencing project. The genomes of six red foxes were sequenced with 6X coverage each and aligned against the dog genome (CanFam3.1). A significant increase in the number of sequencing reads was observed in a ~206 kbp region on dog chromosome 13 (CFA13) containing a partial sequence of the *C-KIT* gene (chr13: ~47,122-47,328 kbp) (Figure 1). The region corresponding to the *C-KIT* promoter, exon one, and part of intron one did not show a read depth increase [48]. The increase in read depth in the region including the partial sequence of *C-KIT* is likely caused by the presence of this region on fox B chromosomes. The depth of reads in this region varied among individuals, suggesting that sequenced individuals carried different numbers of B chromosomes in their genomes. An additional confirmation of the presence of the *C-KIT* region on fox B chromosomes was obtained through sequencing a red fox, flow sorted, B chromosome specific DNA library (Degenerate Oligonucleotide Primed PCR) ([25,52], Makunin et al., in preparation).

These novel sequencing data suggest that the *C-KIT* copy on B chromosomes of the red fox is not translated or is not fully functional. The results partly disagree with previously published data reporting the presence of a full B chromosomal copy of the gene. B specific library sequencing has revealed that total genomic DNA contamination occurs in virtually all flow sorted samples and there is a need for statistical estimates to make conclusions about the presence or absence of specific DNA segments. Therefore, the data on PCR assisted mapping of chromosome specific libraries should be supplemented by other approaches to ensure the results are correct.

Numerous protein coding sequences on fox B chromosomes were discovered using cDNA selection strategy. Briefly, DOP-PCR amplified libraries obtained from sorted fox B chromosomes were used as probes in SHAC (Selection of Hybrids by Affinity Capture) to enrich total fox cDNA with B-specific transcripts. Sequencing of about 40 randomly selected clones of enriched cDNA libraries resulted in the identification of several new putative regions homologous to autosomes. To confirm the presence of the respective sequences on B chromsomes, canine-characterized BACs, containing homologous genes, were localized on fox and raccoon dog B chromosomes using FISH (fluorescent *in situ* hybridization) [53]. These data confirmed the presence of *C-KIT* and revealed over a dozen new autosomal protein-coding gene containing regions on canid B chromosomes (Table 1). Segments of 10 autosomes were discovered on B chromosomes of Canidae, seven of which were present on fox Bs, five on Chinese raccoon dog Bs, and only a single one on Japanese raccoon dog Bs. One of the largest regions, associated with the *LRIG1* (leucine-rich repeats and immunoglobulin-like domain protein 1) gene, showed homology to a 601 kbp interval in the dog and was represented in six overlapping BAC clones. This region was identified only in Chinese raccoon dog B chromosomes. Notably, among ten B chromosomal regions, at least four contained proto-oncogenes: *C-KIT*, *LRIG1*, *RET* (receptor tyrosine kinase), *CTNND2* (cadherin-associated protein) or a tumor suppressor gene, *LRP1B* (LDL receptor related protein 1B) [53].

**Table 1:** **The list of the genes localized on vertebrate B chromosomes**

**Gene**	**Species**	**Function (from GO)**	**References**
*C-KIT* (v-kit Hardy-Zuckerman 4 feline sarcoma viral oncogene homolog)	*Vulpes vulpes*	Protooncogene, encoding a type 3 transmembrane receptor	[40,51,53]
	*Nyctereutes procynoides procynoides*		
	*N. p. viverrinus*		
*KDR* (kinase insert domain receptor)	*N. p. procyonoides*	Protooncogene, encoding a tyrosine kinase receptor	[51]
	*N. p. viverinus*		
*RET* (ret proto-oncogene)	*N. p. procyonoides*	Protooncogene, encoding a tyrosine kinase receptor	[53]
*LRIG1* (leucine-rich repeats and immunoglobulin-like domains 1)	*N. p. procyonoides*	Tumor suppressor gene	[53]
*LRP1B* (low density lipoprotein receptor-related protein 1B)	*V. vulpes*	Tumor suppressor gene, encoding a low density lipoprotein (LDL) receptor	[53]
*CTNND2* (cadherin-associated protein)	*V. vulpes*	Protooncogene, encoding a adhesive junction associated protein	[53]
*IHHB* (Indian hedgehog homolog b)	*Lithocromis rubripinnis*	Embryonic morphogenesis regulation	[59]
Lysosomal alpha-mannosidase	*L. rubripinnis*	Exoglycosidase	[59]
*Rnasel* 2 (Ribonuclease-like 2)	*L. rubripinnis*	Ribonuclease	[59]
VPS10 domain receptor protein SORCS 3–like	*L. rubripinnis*	Neuropeptide receptor	[59]
Ryanodine receptor–like unnamed protein	*L. rubripinnis*	Calcium channels	[59]
*TNNI3K* (TNNI3 Interacting Kinase)	*Capreolus pygargus*	Protein serine/threonine kinase activity	[55]
*FPGT* (Fucose-1-phosphate guanylyltransferase)	*C. pygargus*	Guanylyltransferase	[55]
*LRRIQ3* (Leucine-Rich Repeats And IQ Motif Containing 3)	*C. pygargus*	Not clear	[55]

Only the *C-KIT*-containing region (CFA13) was found to be present on B chromosomes of all three canid species (Table 1). A second CFA13 region, located ~12.5 Mbp from the *C-KIT* gene in the dog genome, was present on both fox and Chinese raccoon dog B chromosomes. All other regions were found to be species-specific.

**Figure 2:**
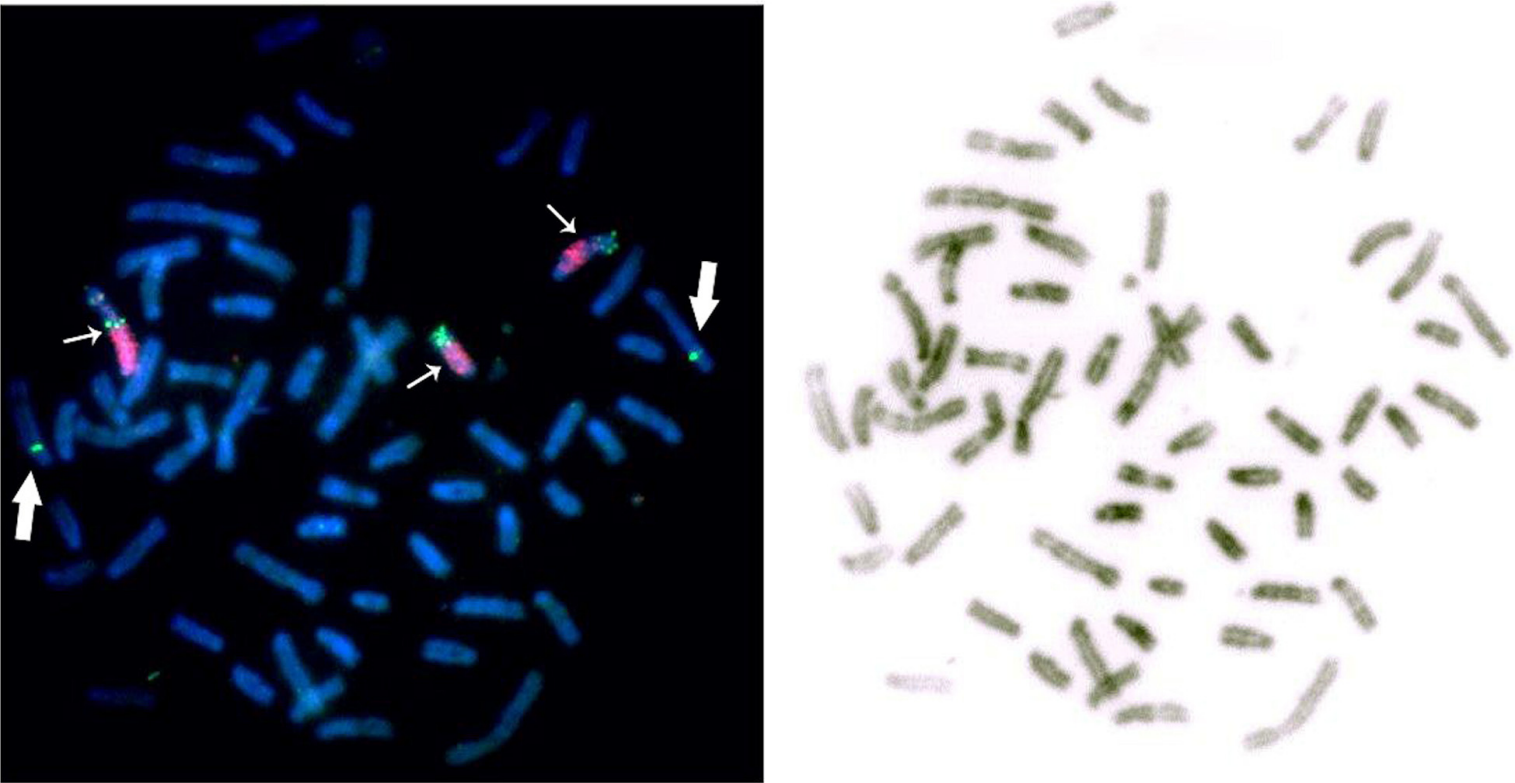
**The localization of the** ***cKIT*** **containing canine BAC 265 L-22 (green) and a probe specific for the proximal region of Japanese raccoon dog B chromosomes (red) on Chinese raccoon dog B chromosomes.** Small arrows indicate B chromosomes; large arrows indicate chromosome 6, where the original copy of *cKIT* is located.

Although FISH analysis revealed the presence of multiple genomic regions on B chromosomes, the exact copy number of duplicated segments remained unclear. In our previous study we obtained region-specific libraries from B chromosomes of the Japanese raccoon dog by microdissection [25]. Using these probes in FISH experiments we showed a significant homology and similarity in DNA content between the B chromosomes of Chinese and Japanese raccoon dogs (our unpublished data, Figure 2). However, many autosomal segments identified in Chinese raccoon dog Bs by BAC mapping were not found in Japanese raccoon dog Bs [53], which may indicate that the FISH results mostly demonstrated repetitive sequence content. Thus, additional experiments are needed to identify the missing content of the rather large Japanese raccoon dog B chromosomes.

Taken together, these findings confirmed the hypothesis of the autosomal origin of B chromosomes in canids and raised questions about the mechanisms preserving the striking conservation of B-chromosomal sequences, since the common content of canid Bs might presume their common origin and 16 million years of independent evolution [40,54]. An alternative explanation for this phenomenon is based on the possibility that some sequences are re-used for B chromosome formation in various lineages independently. For example, we recently discovered the presence of the *C-KIT* gene on Bs of the cervid species brown brocket deer (*Mazama gouazoubira*) (Makunin et al., in preparation).

## B chromosomes of the Siberian roe deer

In the *Cervidae* family, B chromosomes were identified in the genomes of two genera: *Mazama* and *Capreolus*. A recent study of B chromosome content and activity in the Siberian roe deer (*Capreolus pygargus*) was the first to indicate that some genes on B chromosomes are transcribed in vertebrates [55]. A candidate 2 Mbp region including *FPGT*, *LRRIQ3* and *TNNI3K* genes was found on B chromosomes of Siberian roe deer by a B-specific cDNA selection approach (see Table 1 for gene details). The presence of these three genes on B chromosomes of this species was further confirmed using FISH with bovine BAC clones, PCR-assisted mapping, real-time PCR and B-specific library sequencing. Interestingly, these genes were shown to be unevenly amplified among Bs of Siberian roe deer. Sequencing of B-specific and autosomal copies of the three genes revealed mutations specific for B chromosomes. One of these B-specific mutations was found in a transcript of the *FGPT* gene, thus demonstrating transcription of a B-specific gene copy. These novel data generally challenge the view of totally silent B chromosomes in vertebrates, and promise similar new discoveries in other species.

## B chromosomes of cichlid fishes

In cichlid fishes, B chromosomes have been identified in seven South American and fourteen African species [56-60]. Cichlids represent a model for adaptive radiation and extensive genomic resources are available for several representatives of the group: genetic and physical maps for several species and sequenced genomes of four species (*Astatotilapia burtoni*, *Pundamilia nyererei*, *Metriaclima zebra* and *Neolamprologus brichardi*). Recent studies in two cichlid species provide new insights on the genetic content, origin, and functions of B chromosomes.

### Genes on B chromosomes of *Lithocromis rubripinnis*, a cichlid species of Lake Victoria

In one of populations of *Lithocromis rubripinni*s, a cichlid species from Lake Victoria [59], only females carried B chromosomes. Experimental breeding of B-lacking individuals produced progeny with a sex ratio close to 1:1, while but B-carrying animals had a much higher proportion of female offspring (up to 100%). The authors suggested that in this case sex determination was directly linked to B chromosomes.

A library of BAC clones from a B-carrying *L. rubripinni*s individual was created and B-chromosome-specific clones were identified based on differential hybridization with genomic DNA of individuals with (B+) and without (B-) B chromosomes. The analysis of B chromosome specific DNA from BAC clones revealed a B-specific repeat and fragments of five protein-coding genes. These genes were proposed to be functional as no nonsense mutations were found, but none of these genes was thought to be directly involved in sexual development (Table 1). One of the genes, *IHHB* (indian hedgehog homolog b), was extensively amplified – about 40 copies were found in the *L. rubripinnis* genome by qPCR.

Using FISH for BAC-clone localization, the authors showed that Bs were homologous to the short arm of chromosome 1. This region arguably contains loci responsible for sex determination. Although the origin of some B chromosomes from sex chromosomes was proposed earlier (reviewed in [8]), the extraordinary feature of the role of B chromosomes in sex determination was demonstrated here for the first time.

### DNA content of B chromosomes in the cichlid astatotilapia latifasciata

Another recent study investigated the genetic content of B chromosomes in *Astatotilapia latifasciata*, a cichlid species from lake Nawampasa (Lake Kyoga system, Africa), using a whole genome sequencing approach [61]. Briefly, Illumina HiSeq reads (23X coverage for an individual carrying B chromosomes (B+) and 31X coverage for an individual without B chromosome (B-)) were mapped to the *Metriaclima zebra* reference genome. Genomic regions specific to B chromosomes (B-blocks) were identified based on difference coverage of normalized B+ and B- libraries. To test these results, DNA from a microdissected B chromosome was amplified and sequenced using the Roche 454 platform. Reads were assembled in contigs and mapped to the same *M. zebra* reference genome after filtering out human contaminants.

The resulting positions of B-specific sequences were in good agreement between the two methods. B-blocks were distributed among most linkage groups of *M. zebra*, but the largest blocks were identified in linkage groups 1, 3 and 9 (165, 177 and 146 kbp, respectively). Interestingly, these blocks include genes previously identified on B chromosomes in cichlids from Lake Victoria (*Lithochromis rubripinnis*) [59,60]. Based on this fact, the authors proposed a common origin of B chromosomes in these species. The study of gene content of B-blocks has shown that only 3.2% of 5,858 putative B genes (including processed pseudogenes) had over 50% of their sequence present on B chromosomes. FISH mapping and qPCR confirmed B-specific localization and copy number changes of the *HPRT* gene. Among genes with high integrity, the authors highlighted several genes involved in chromosomal segregation: proteins involved in microtubule organization (*TUBB1, TUBB5*), kinetochore structure (*SKA1, KIF11, CENP-E*), recombination (*XRCC2, SYCP2, RTEL1*) and progression through the cell cycle (*Separase*, *AURK*). Trancriptome analysis revealed that B-chromosomal copies of the *Separase, TUBB1 and KDF11* genes are expressed, but evolve under neutral selection.

Authors proposed a scenario of B chromosome origin: segmental duplications including the centromeric region with subsequent formation of an isochromosome, amplification of sequences and introgression of additional segments from the main genome through transposable and retrotransposable elements and ectopic recombination.

## Genes on B chromosomes of the Amazon molly *Poecilia formosa* (Poecillidae, Cyprinodontiforme)

In the Amazon molly *Poecilia formosa* (Poecillidae, Cyprinodontiforme), expression of genes affecting pigmentation was shown to be linked with the occurrence of B chromosomes in the karyotype [62]. Moreover, additional chromosomes of this species harbor an uncharacterized locus that confers a higher susceptibility to pigment cell tumors [63]. In a recent study the presence of a unique region on B chromosomes of this species was shown using AFLP (amplified fragment length polymorphism). The Southern hybridization confirmed that this region is represented by a single copy and is conserved within the *Poecilia* genus [64].

## Insights from studies of B chromosomes in non-vertebrate organisms

The overall picture of nature of supernumeraries would be incomplete without mentioning numerous studies of B chromosomes in taxa beyond vertebrates. Because of the scope of our review we will only briefly summarize the results of some outstanding B chromosome studies in three groups: fungi, flowering plants, and orthopterans.

The presence of genes on supernumerary chromosomes was first predicted in antibiotic resistance studies of the fungus *Nectria haematococca* [65,66]. Later studies further characterized a gene located on additional karyotypic elements of *N. haematococca* and provided evidence of its expression [67]. A recent *N. haematococca* genome sequencing project revealed that supernumeraries are enriched in genes which potentially expand the ecological niche of the host.This study also revealed B chromosome specific genes similar to those from different fungi species, and even some regions supposedly originating from horizontal gene transfer [68].

A number of B chromosome studies have been performed in plants. B chromosomes are present in hundreds of flowering plant species, including economically important ones such as rye, oat and maize [9,69]. A map of high-copy repetitive sequences in B chromosome was constructed in rye [70], and regions connected with meiotic non-disjunction [71] and expressed pseudogenes [72] were identified. High-throughput sequencing of flow-sorted B chromosomes was performed in rye [73] and maize (Blavet et al., unpublished data) in both cases revealing that B chromosomes include extended genomic regions with some genes present. The presence of transcriptionally active genes on some oat (*Avena saliva* L.) B chromosomes was shown by their effects on host resistance to rust [74].

Another group with actively studied B chromosomes is the Orthoptera family in insects. While many studies are devoted to B chromosome evolution and population dynamics, some interesting results concerning B chromosome molecular composition were obtained. In the migratory locust, copies of ribosomal genes were found to be active on B chomosomes [75]; divergence between H3 and H4 histone gene copies located on B chromosomes and chromosome 8 was used to estimate the age of supernumeraries in this species [22].

## Possible effects of B chromosomes on fitness

The application of current methods of molecular biology such as FISH, sequencing, real-time PCR, and AFLP allowed the localization of specific sequences, including protein-coding genes (listed in Table 1), on B chromosomes of several vertebrate species. These sequences seem to be amplified on B chromosomes [53,59,61]. The presence of genes involved in various processes like oncogenesis [53,64], sex determination [59] and cell division [61] on vertebrate B chromosomes indicates that supernumeraries may play a particular role in the development and overall fitness of host organisms. High throughput sequencing technologies provide an intriguing possibility for obtaining an overall picture of B chromosome sequence, expression and epigenetic status [61,73]. Investigation of B chromosomes using these methods will lead to insights into chromosome structure, function and evolution, and may also provide an impetus for constructing chromosome-based vectors, namely mammalian artificial chromosomes (MACs).

## Role of B chromosomes in the evolution of genomes

Little is yet known about the role of B chromosomes in the evolution of genomes. How do they originate? Why do they occur more frequently in some species than in others? Are they short term events or do they persist in genomes for a long time? Further analysis of the molecular content of the B chromosomes can answer some of these questions.

The mapping of B chromosomes in the vertebrate species considered above [40,53,55,59,61,64] revealed the presence of large autosomal regions containing protein-coding genes. Regions mapped on B chromosomes show a significant similarity with homologous autosomal regions ([59,64] and our unpublished data). Therefore we can conclude that most B chromosomes contain segment duplications [76].

Segment duplications originate as a result of improper recombination. In most cases they are tandemly arranged and can be subsequently transposed to distant genomic regions. It has been shown that even partially duplicated genes can be functional [77], indicating that incomplete gene copies on B chromosomes may also play some role in cell metabolism. Moreover, B-chromosomal and autosomal copies of genes are highly homologous, which can be treated as indirect evidence of their functionality [55,59,64]. Amplified copies of genes located on B chromosomes, if they are expressed, can affect the host organism. For example, changes in the copy number of functional *C-KIT* genes may lead to changes in pigmentation, gametogenesis disorders or various cancers [78-80]. Duplicated genes on B chromosomes can accumulate mutations, theoretically resulting in new functions or domain fusion of previously unassociated genes, similar to the effects of segment duplications described above.

A significant number of genes amplified on B chromosomes are connected with oncogenesis [40,53,64]. In mammals, oncogene amplification leads to the formation of homogeneous staining regions (HSRs) and double minute (DM) chromosomes [11]. HSRs represent amplified chromosome regions containing oncogenes, and DMs are small circular autonomic acentromeric chromosomes with the same content. The similarity between Bs and HSRs/DMs is not limited to their genetic content. B chromosomes may also lack typical centromeric DNA (alphoid satellites) (unpublished data by DV Yudkin) and behave irregularly during cell divisions. Similar properties are characteristic for DMs [81].

HSRs and DMs have been intensively studied. Among other results, various mechanisms have been proposed for their formation: the breakage-fusion-bridge model postulates amplification of genes near chromosomal breakpoints; the translocation-deletion-amplification model posits that HSRs and DMs are formed from breakpoint regions in translocation events; and the deletion-plus-episome model assumes excision of a DNA segment with further circularization, amplification and mutual recombination [82]. Based on the many similarities between Bs and HSRs/DMs, we can propose that some of the mechanisms involved in B chromosome formation may be common with those described for HSRs and DMs.

Recent findings in cattle indicate that color sidedness [83] and gonadal dysgenesis [84] are caused by complex rearrangements (a combination of duplication, translocation and circularization) involving the *C-KIT* gene. Mechanisms of these rearrangements are similar to those proposed for B chromosome (as well as HSR and DM) formation. This may indicate that the genomic regions involved in such rearrangements are inherently unstable in different species and perhaps represent recombination hotspots. Based on this example one may speculate that B chromosomes are likely to originate from regions with a high frequency of illegitimate (non-homologous) recombination or evolutionary breakpoints.

Small supernumerary marker chromosomes (sSMCs) are found in human populations with a frequency of 1/2000. Phenotypic effects of sSMCs vary from normal to serious clinical syndromes. As well as B chromosomes, they can include euchromatic and heterochromatic regions, are mitotically unstable and have predominantly maternal transmission. Interestingly, most sSMCs originate from the the p-arm of chromosome 15 [85]. Although in many respects sSMCs and Bs are similar, the latter acquired a drive mechanism allowing for accumulation through generations in populations. Thus sSMCs can serve as a model of the early evolution of Bs [12].

In summary, research on the genetic content of B chromosomes enables discussion of their origin and evolution, as well as of the molecular mechanisms of irregular behavior in cell division coupled with persistence over many generations. Little is yet known about the interactions between B chromosomes and the main genome; Bs are a separate yet communicating pool of genetic diversity which can increase the variability of the main genome. Further research carefully examining the borders of amplification regions and comparing them with autosomal homologs will shed light on rearrangement events that took place millions of years ago.

## Conclusion and perspectives

The emerging science of genomics allows large amounts of information about the structure and function of specific genomic regions to be obtained. Detailed characterization of vertebrate B chromosomes by modern technologies might reveal new important functional elements localized in supernumeraries, help estimate their possible effect on host organisms, and suggest new scenarios and mechanisms of vertebrate genome evolution. Indeed, recently obtained data clearly indicate that the role of B chromosomes in evolution and their influence on the host were underestimated. Future genome projects [86] should consider a possible role of B chromosomes in various cellular and evolutionary processes.
